# Prevalence, Genotypic and Phenotypic Characterization and Antibiotic Resistance Profile of *Clostridium perfringens* Type A and D Isolated from Feces of Sheep (*Ovis aries*) and Goats (*Capra hircus*) in Punjab, Pakistan

**DOI:** 10.3390/toxins12100657

**Published:** 2020-10-14

**Authors:** Mudassar Mohiuddin, Zahid Iqbal, Abubakar Siddique, Shenquan Liao, Muhammad Khalid Farooq Salamat, Nanshan Qi, Ayesha Mohiud Din, Mingfei Sun

**Affiliations:** 1 Key Laboratory of Livestock Disease Prevention of Guangdong Province, Maoming Branch, Guangdong Laboratory for Lingnan Modern Agriculture, Scientific Observation and Experiment Station of Veterinary Drugs and Diagnostic Techniques of Guangdong Province, Ministry of Agriculture, Institute of Animal Health, Guangdong Academy of Agricultural Sciences, Guangzhou 510640, China; lsq6969@163.com (S.L.); nanshanqi@163.com (N.Q.); 2 Department of Microbiology, Faculty of Veterinary and Animal Sciences, The Islamia University of Bahawalpur, Bahawalpur 63100, Pakistan; 3 Department of Pharmacology, Swat Medical College, Marghzar Road, Saidu Sharif, Swat 19200, Pakistan; zahid1.iqbal1@gmail.com; 4 Atta Ur Rahman School of Applied Biosciences (ASAB), National University of Sciences and Technology (NUST), H-12, Islamabad 44000, Pakistan; Abubakars974@gmail.com; 5 The Roslin Institute, University of Edinburgh, Easter Bush, Midlothian EH25 9RG, UK; khalid.salamat@roslin.ed.ac.uk; 6 Department of Biotechnology, Virtual University of Pakistan, 1-Davis road, Lahore 54000, Pakistan; ayesha.mohiuddin@vu.edu.pk

**Keywords:** *C. perfringens*, antimicrobial susceptibility, sheep, goats, toxinotyping, multiplex PCR

## Abstract

*Clostridium perfringens* poses a serious threat to small ruminants by causing moderate to severe enterotoxaemia. Due to its ability to produce a wide arsenal of toxins, it is ranked among the most prevalent and important pathogens in livestock. This study focused on the molecular characterization of different *Clostridium perfringens* types along with their antimicrobial resistance profile. An overall higher prevalence of *C. perfringens* (46.1%) was detected based on mPCR among sheep and goats (healthy and diseased) in the Punjab province, Pakistan. The majority of the isolates were characterized as type A (82%), followed by type D (18%). Among the isolates from diseased sheep and goats, 27% were positive for *cpa*, 49% for *cpa* and *cpb2*, 9% for *cpa* and *etx*, 15% for *cpa*, *cpb2* and *etx*. In the case of isolates from healthy sheep and goats, 59% were positive for *cpa,* 34% for *cpb2* and *cpa*, 4% for *cpa* and *etx*, and 3% for *cpa*, *cpb2* and *etx*. The prevalence of the *beta2* toxin gene in the diseased sheep and goat population was 64% as compared to 37% in healthy animals. All 184 isolates (100%) were sensitive to rifampin and ceftiofur; the majority (57%) was sensitive to teicoplanin, chloramphenicol, amoxicillin, linezolid and enrofloxacin. A lower proportion of isolates (43%) were sensitive to ciprofloxacin and only 14% were susceptible to erythromycin. The findings of this study highlight the higher prevalence of *C. perfringens* in small ruminants and indicate that detailed pathogenesis studies are necessary to understand the explicit role of various toxins in causing enteric infections in sheep and goats including how they might be exploited to develop vaccines against these diseases.

## 1. Introduction

The livestock industry is one of the fastest growing and financially important sub-sectors of the economy in developing countries like Pakistan, accounting for a 60.54% value addition to the Pakistani agricultural sector and contributing 11.22% to GDP. Furthermore, it makes a significant contribution to rural economic growth, where 40% of the population is dependent on livestock [1]. Small ruminants, especially sheep and goats, are required for the subsistence of poor livestock owners and are a main source of meat, milk, manure and skin [2]. Unfortunately, over the last decade, the caprine and ovine industry has significantly declined due to infectious diseases [3]. *Clostridium perfringens* is one of the main pathogens responsible for intestinal infections as well as histotoxic diseases in animals, having a low incidence rate (2–8%) but a very high case fatality rate (100%) [4,5]. It is a gram positive, anaerobic, spore-forming microorganism that produces more than 20 extracellular toxins and hydrolytic enzymes, of which six are currently used for typing [6]. The toxins form diverse combinations and can be divided into types A–G. All types produce α-toxin; in addition, type B produces β- and ε-toxin, type C produces β-toxin, type D produces ε-toxin, type E produces ι-toxin, type F produces enterotoxin and type G produces netB toxin [7,8]. All these potent toxins can act locally or are absorbed in the intestine causing enteric infections, which are generically named as enterotoxemia in sheep and goats. However, the severity of these infections is dependent on the type and combination of various toxins, of which epsilon toxin (type D) is the most frequent cause of enterotoxemia in sheep and goats [9]. *C. perfringens* type A is involved in acute enterotoxemia in the goat kids [10]. Type D is associated with “pulpy kidney disease” and dysentery in sheep [11]. Epsilon toxin, present in *C. perfringens* type B and D, is mostly involved in disease pathogenesis [7]. Epsilon toxin in goats causes enterocolitis, but in sheep it can also cause abnormal effects on the brain and lungs. The difference in pathogenesis between the two species is due to the different mechanisms by which epsilon toxin modifies water and ion transport in the intestines of sheep and goats [12,13,14]. In developed countries, *C. perfringens* type F food poisoning is a commonly reported food-borne illness [15]. Strains carrying the CPE gene (*cpe*), isolated from both humans and animals, produce spores in the intestinal tract. These spores produce enterotoxin (CPE), which is responsible for symptoms such as diarrhea [16,17].

Although *C. perfringens* can exist as part of the normal intestinal microflora, under certain conditions where the physiological balance of the intestine is altered, abnormal proliferation of this microorganism can result in the production of a wide variety of toxins [18]. These toxins either act locally or are absorbed into the general circulation, resulting in severe effects on the host [19]. Accurate typing of the toxin is very important in disease diagnosis [20]. Traditionally, typing of *C. perfringens* strains has been performed using a toxin neutralization test with the appropriate antisera in laboratory animals such as guinea pigs [21]. An ELISA (enzyme-linked immunosorbent assay) based method can also be used for typing *C. perfringens* isolates. However, these methods are often time-consuming and are not always able to identify a range of different toxins, thereby limiting subtyping options [22]. In addition, special culture methods are required to induce sporulation, which aids in the identification of enterotoxin producing strains [23]. Genotyping of *C. perfringens* isolates, however, can overcome this problem and has become the standard for toxinotyping of *C. perfringens*. Polymerase chain reaction (PCR) protocols, especially multiplex PCR assay, have been established for simultaneous detection of these toxin genes (*cpa, cpb, etx, iap, cpe* and *cpb2*) [24,25,26,27].

Although vaccines and different antimicrobial agents are used to control enterotoxaemia [28], the extensive use of these agents to increase the growth rate of livestock animals and treat gastrointestinal infections likely serves as a way of transmitting antimicrobial-resistant genes or microbes into the human food chain [29,30]. The emerging problem of antimicrobial resistance between pathogenic and commensal bacteria is also of concern [31]. There are very few reports on the antibiogram of *C. perfringens* isolates recovered from sheep and goats in Pakistan. More comprehensive epidemiological information pertaining to *C. perfringens* type A and D in these animals is also lacking. As such, the aim of the present study was the isolation, phenotypic, genotypic and molecular typing of *C. perfringens* type A and D isolates from diseased and healthy sheep and goats in the Punjab province of Pakistan, as well as the analysis of their antimicrobial susceptibility patterns.

## 2. Results

### 2.1. Isolation and Identification of C. perfringens

A total of 201 samples (50.31%) from both sheep and goats were found positive and exhibited typical black colonies on tryptose sulphite cycloserine agar plates. Most of isolates showed the presence of Gram-positive rods and possessed subterminal spores. On blood agar, characteristic beta hemolytic colonies with double zone of hemolysis were observed. The microorganism produced an opaque halo around colonies on egg yolk agar, confirming it as *C. perfringens* whereas most other Clostridia are reported to be lecithinase negative. All culture positive isolates were further resolved through biochemical differential tests and sugar fermentation reactions. Almost all isolates fermented glucose, maltose, sucrose, lactose, mannose and trehalose, while arabinose, cellobiose, mannitol, melezitose, salicin, xylose, sorbitol and rhamnose were not fermented by *C. perfringens* isolates (Table 1). Two sugars behaved differently in different isolates. Glycerol was fermented by one group of isolates while the other group did not ferment it. Variations were also seen in the case of raffinose sugar, i.e., most of the isolates did not ferment this sugar while only a few fermented it. There were also slight variations in the biochemical properties of different isolates. All isolates hydrolyzed gelatin and were negative for urease production. In addition, very few (2%) isolates hydrolyzed esculin. The indole test was negative in all of the isolates; however, there was one isolate that behaved differently (i.e., indole was positive). Biochemical characterization, therefore, differentiated the isolates into four major groups; these are given in Table 2. It was found that 195 (48.9%) culture isolates belong to *C. perfringens* on the basis of biochemical characterization.

### 2.2. Toxinotyping of Recovered C. perfringens Isolates

**Figure 1:**
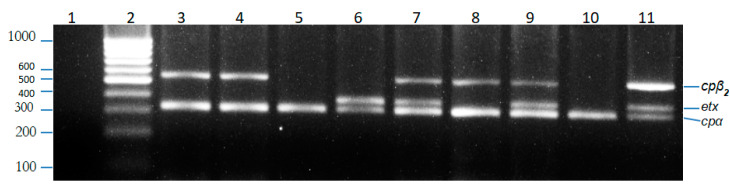
Agarose gel electrophoresis of PCR products. Lanes: 1, negative control (no template DNA); 2, 1000 bp ladder; 3,4,8, *C. perfringens* isolate (genotype A, β2); 5,10, *C. perfringens* isolate (genotype A); 6, *C. perfringens* isolate (genotype D); 7,9, *C. perfringens* isolate (genotype D, β2); 11, Positive control.

All 195 biochemically characterized isolates of *C. perfringens* were examined for *alpha, beta, epsilon, iota, beta2* and enterotoxin gene by using the multiplex PCR technique. The alpha toxin gene *(cpa)* was found to be present in 184 out of 399 (46.1%) isolates by simple PCR. These 184 isolates were further genotyped using mPCR to further identify them as either type A or type D; and categorized on the basis of presence of major toxin genes. Type A isolates had an *alpha* gene while type D isolates had *alpha* and *epsilon* genes. Neither *beta* nor *iota* genes were present in any of the genotyped isolates. In addition to *alpha* and *epsilon* genes, *beta2* was also found in both type A and type D isolates. Eighty-two percent of the isolates from sheep and goats belonged to *C. perfringens* Type A while 18% of the isolates were *C. perfringens* type D (Figure 1).

Among the isolates from healthy sheep and goats, 59% were positive for *cpa*, 34% for *cpb2* and *cpa*, 4% for *cpa* and *etx,* and 3% for *cpa*, *cpb2* and *etx.* In the case of isolates from diseased sheep and goats, 27% were positive for *cpa,* 49% for *cpa* and *cpb2*, 9% for *cpa* and *etx*, and 15% for *cpa*, *cpb2* and *etx.* Therefore, the percentage of isolates that were positive for *cpb2* was significantly higher in the diseased population (64%) of sheep and goats as compared to the healthy population (37%). As far as the presence of *etx* gene was concerned, 24% of isolates from the diseased population had the *etx* gene, compared to 7% from the healthy population. The percentage of *cpa* and *cpb2-*positive isolates from diseased animals (49%) was significantly higher than that of healthy animals (34%). No isolates from both populations were found to carry *cpb, iap* and *cpe* (Table 3 and Table 4).

### 2.3. Antimicrobial Susceptibility Patterns

**Figure 2:**
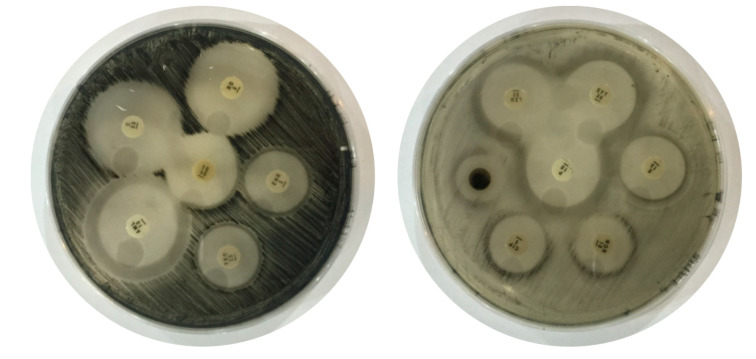
Antibiotic susceptibility testing of *Clostridium perfringens* against different drugs.

A total of 184 *C. perfringens* type A and D isolates confirmed by mPCR were used for in vitro antimicrobial susceptibility testing (Figure 2). All the *C. perfringens* isolates showed 100% susceptibility to rifampin and ceftiofur, while teicoplanin and enrofloxacin showed “significant susceptibility” against 57% of the *C. perfringens* isolates. Teicoplanin showed “good susceptibility” against 29% of the isolates while enrofloxacin showed good susceptibility against 43% of the isolates of *C. perfringens*. Chloramphenicol, amoxicillin and linezolid showed significant susceptibility against 57% of *C. perfringens* isolates. Erythromycin showed significant susceptibility to 14% isolates while good susceptibility was observed against 72% of the isolates. Norfloxacin showed “moderate susceptibility” against 100% of the isolates. Ciprofloxacin showed significant susceptibility against 43% of the isolates while 29% and 28% of the isolates showed good and moderate susceptibility, respectively. All *C. perfringens* isolates showed 100% resistance against neomycin and 72% resistance to tetracycline as shown in Table 5.

## 3. Discussion

*Clostridium perfringens* ranks among the most important pathogens in livestock and humans, and it causes both histotoxic disease and infection originating in the intestines. Toxins produced by *C. perfringens* types are responsible for enteric diseases in sheep and goats and it has been suggested that economic losses due to *C. perfringens* infections may be a result of all seven types of the bacterium [32,33]. There may be geographical differences in the prevalent types of the bacterium; also types may vary depending on the animal species in the area [34]. In the present study, the biochemical results were similar to previously reported findings, with few exceptions [35,36]. Very few isolates hydrolyzed esculin and glycerol sugar was weakly fermented in some isolates; in some cases there was no fermentation. Raffinose was only fermented in some isolates [37]. A variation was noted during indole production reaction, although only a single isolate was found as indole positive. This variation is significant as it has not been previously reported in the scientific literature. A study conducted in 1942, however, supports this finding [38].

In this study, *C. perfringens* type A was present in 82% of samples collected from both healthy and diseased sheep and goats. Different studies have reported *C. perfringens* type A in 51–87% of samples taken from soil and healthy ruminants [39,40,41]. *C. perfringens* type A has also been reported as the most prevalent type in North America [42]. Another study conducted in Saudi Arabia indicated that *C. perfringens* type A was the most prevalent type in sheep, calves and chicken [43]. Unfortunately, there are very few authentic reports citing the prevalence of *C. perfringens* in Pakistani sheep and goat populations. *C. perfringens* type D causes diseases in numerous animal species; however, it is more prevalent in sheep and goats. The results of our study indicated that *C. perfringens* type D is prevalent in both healthy and diseased animals. About 18% of isolates from both healthy and diseased sheep and goats belong to *C. perfringens* type D. In ruminants, this type is normally present in the intestine even though different studies have isolated this type in less than 20% of the animals and on some farms, no type D microorganism was found at all [41]. In this study, 5 type D isolates out of 68 (7%) samples were found in healthy animals. Of these 5 samples, 2 were isolated from goat and the other 3 isolates belonged to sheep. Type E infections in domestic animals were considered as a rare occurrence. Likewise, no isolates in our study from healthy or diseased animals were found to be *C. perfringens* type E. In numerous epidemiological studies, *C. perfringens* types possessing the *cpb2* gene have been isolated from cattle, horses, pigs, sheep, goats, poultry, fish, carnivores, domestic wildlife species and humans [44,45]. In this study, the prevalence of *beta2* toxin gene was found to be significantly higher in diseased sheep and goats. In diseased animals, the *cpb2* gene was found in 64% of the isolates, compared to 37% in healthy animals. The high prevalence of the *cpb2* gene in isolates from animals with enteric problems is also consistent with previous studies from different parts of the world [46].

The investigations carried out in control group (healthy sheep and goats) revealed that the most prevalent type in these animals was type A (93%). This is because they have *cpa* only, while the other 7% of isolates have epsilon toxin gene in addition to the alpha gene, thus, they belong to type D. In one study conducted in Turkey, 61 *C. perfringens* isolates from healthy sheep were examined by multiplex PCR; of these, 95% belonged to type A and the remaining 5% were type D [47]. These results are similar to those found in previous studies [48,49,50]. A study investigating the predominant flora of ostrich intestine reported type A as the most prevalent type, while in the investigation of fecal samples of diseased (Enteritis, Enterotoxemia) sheep and goats, *C. perfringens* type A was found in 76% of isolates. Similarly, a study conducted in 2012 in Saudi Arabia reported type A in 73.5% of the genotyped isolates [51]. Type A has also been reported as a predominant type in sheep with enterotoxemia [47]. Based on our findings, besides type A, *C. perfringens* type D was found in 24% of isolates from diseased sheep and goats. Reports from various studies around the world have found that the prevalence of type D enterotoxemia ranges from 24.13–100% [32]. In Turkey, the prevalence of type D enterotoxemia in diseased animals was reported to be from 38.63–50% [49].

The present study ascertained the distribution of *C. perfringens* toxin types in sheep and goat populations in relation to health status. The predominance of the *alpha* toxin gene (*C. perfringens* type A) is in accordance with the high distribution of this toxin type in neighboring countries like China, India and Bangladesh [52,53]. The high prevalence of *alpha* and *beta2* genes also signifies the need to incorporate these strains into the vaccine that is currently used against *C. perfringens* infections, which contains only type D strain, and also suggests the use of an oil based/montanide adjuvanted vaccine, which was proven successful against other clinically important diseases of livestock [54]. In addition, this study is the first of its kind to report the presence of epsilon toxin gene (*etx*) in the feces of healthy sheep and goats raised in Pakistan. 

In Pakistan, there are very few reports on the antibiotic resistance patterns of *C. perfringens* type A and D isolated from an animal source. The present study revealed that all isolates were found to be susceptible to penicillin, as mentioned in different studies reported from other countries [55,56]. In addition to penicillin, all isolates were also found to be susceptible to rifampin and ceftiofur. These results were in agreement to those reported in Brazil and Thailand [57,58]. Amoxicillin and enrofloxacin showed high in vitro activity against 57% of isolates and 60–70% inhibition against the remaining 43% of isolates. Another study showed that all isolates from commercial turkeys were highly susceptible to amoxicillin and enrofloxacin [55]. The teicoplanin activity was also found to be in accordance with the results described against intestinal anaerobic bacteria [59].

Norfloxacin, neomycin and tetracycline were least active against *C. perfringens* isolates from sheep and goats. The poor efficacy of these antibiotics has also been previously reported in piglets [57]. Reduced susceptibility to tetracycline detected in *C. perfringens* isolates across all host species is consistent with the findings of several studies from various countries [60,61]. In this study, 72% of isolates were found to be resistant to tetracycline, which is more than previous findings [62]. This higher incidence of tetracycline resistance in *C. perfringens* isolates is the result of the excessive use of this antibiotic in the sampling areas, often following poor or incorrect veterinary advice. In the present study, linezolid and chloramphenicol showed significant susceptibility against 57% of *C. perfringens* isolates while ciprofloxacin and erythromycin showed intermediate susceptibility. Previous studies have indicated that almost all isolates were susceptible to linezolid, chloramphenicol while resistance against erythromycin antibiotic was reported for fewer isolates [56,62,63,64].

Our findings showed that tetracycline is a poor treatment choice for *C. perfringens*-associated infections due to the decreased susceptibility among *C. perfringens* isolates. Therefore, the use of tetracycline, particularly at low doses, could select for tetracycline resistant strains and result in the transmission of tetracycline resistance.

## 4. Conclusions

In Pakistan, enteric diseases in small ruminants are causing annual losses of billions of rupees to the sheep and goat industry. The high distribution of toxins in strains from clinically infected sheep and goats also highlights their role in producing diseased conditions. Consequently, these initial findings could be further used for epidemiological investigations, prophylaxis plans and control strategies by formulating appropriate vaccines.

## 5. Materials and Methods

### 5.1. Sample Site and Isolation Source

A total n = 399 samples were collected aseptically in sterile plastic bags from sheep and goats in selected districts (Faisalabad, Okara, DG Khan, Muzaffargarh, Bhakkar, Layyah) in the Punjab province, Pakistan over a one year period using a convenient random sampling technique. Fecal samples were collected from healthy and diseased animals following the inclusion and exclusion criteria. Healthy sheep and goats included animals that were assessed as healthy with no history of diarrhea and with normal fecal consistency. In addition, these animals had not received any other medical treatment in the last month. Diseased animals included sheep and goats suffering from enteric disease (having diarrhea or history of diarrhea) which may include fever, abdominal discomfort, lack of appetite and a history of weight loss. Vaccinated animals (alum precipitated enterotoxemia vaccine) were also included according to their health status either in the healthy or diseased group. Animals undergoing any medical treatment in the last month were excluded from the study. Collected samples were transported immediately to the laboratory under refrigerated conditions.

### 5.2. Isolation of C. perfringens

The fecal samples were diluted in phosphate buffered saline (PBS) 1:10 and placed in a water bath for 10 min at 80 °C to kill the non-spore forming microorganisms. The processed samples were sub-cultured in reinforced clostridial media (RCM) and then inoculated on tryptose sulfite cycloserine agar (TSC) and 5% blood agar plates. The inoculated plates were kept in anaerobic jars at 37 °C for 24 h. Anaerobiosis was created by using an anaerobe sachet. After initial identification based on characteristic colony morphology and Gram staining, the colonies presenting *C. perfringens* characteristics were streaked on egg yolk agar in order to confirm the lecithinase and lipase activity. Further biochemical characterization was carried out using API 20A kits (bioMérieux, Marcy l’Étoile, France).

### 5.3. Molecular Typing of C. perfringens Through Multiplex PCR (mPCR)

Following biochemical characterization, isolates found positive for *C. perfringens* were subjected to molecular typing for identification of the prevalent toxin types. Molecular typing of *C. perfringens* was performed by multiplex PCR technique. Accordingly, previously developed primers *cpa, etx, cpb, iap, cpe* and *cpb2* (Table 6) were used simultaneously for their ability to identify *C. perfringens* types [65,66,67].

Genomic DNA was extracted from all *C. perfringens* isolates using a manual method [34]. Colonies from 12–18 h pure culture of *C. perfringens* were suspended in 1 mL distilled water (about 10^6^ cells per mL). Centrifugation was done at 6000× *g* for 5 min. The supernatant was discarded and cells were resuspended in 200 μL cold Tris EDTA (TE) Buffer. DNA was extracted using EZ-10 Spin Column DNA Gel Extraction Kit (Bio Basic Inc., Toronto, Canada). Multiplex PCR was performed in a thermal cycler with final volume of 25 µL for each reaction mixture. The reaction mixture contained 1.25 U Taq DNA polymerase, 1X PCR Buffer, 4 mM MgCl_2_, 250 µM dNTPs, 0.12 µM forward and reverse primers of alpha, epsilon, beta, iota and entero gene, 0.16 µM forward and reverse primer of *beta2* gene, 2 µL of sample DNA (150–200 ng/µL). The PCR conditions consisted of a pre-denaturation phase at 95 °C for 10 min and 40 cycles of 94 °C for 45 s, 55 °C for 90 s, 72 °C for 90 s followed by 72 °C for 10 min. The PCR product was electrophoresed on 1.5% agarose gel. DNA bands were observed under UV transillumination and photographed using Alpha Imager Mini Imaging System (San Jose, CA, USA).

### 5.4. Antimicrobial Susceptibility Testing

Antibiotic susceptibility assays were performed according to the disk diffusion method described in [68]. Thirteen different antibiotics were selected on the basis of clinical relevance and veterinary farm practices, belonging to different antimicrobial groups. The antibiotic discs used (Oxoid, Basingstoke, UK) were as follows: amoxicillin (10 µg), ceftiofur (30 µg), chloramphenicol (30 µg), ciprofloxacin (5 µg), enrofloxacin (5 µg), erythromycin (15 µg), linezolid (30 µg), neomycin (10 µg), norfloxacin (10 µg), penicillin G (10 µg), rifampin (5 µg), teicoplanin (30 µg) and tetracycline (30 µg). Perfringens agar medium (Oxoid, UK) was used as a growth medium for *C. perfringens*. A single colony from a 24 h fresh culture was dispensed in sterilized saline solution by using a sterilized inoculating loop. After thorough mixing, the turbidity of the cultures was compared with standard McFarland solution. If the broth was more turbid, they were adjusted through the addition of normal saline. Bacterial lawn was made on the petri plates by dipping sterile cotton swab in bacterial inoculum. Antibiotic discs were placed on the inoculated plate at an appropriate distance from each other. Penicillin was taken as a positive control. The clear zones of inhibition appeared around the discs of antibiotics after 24 h of incubation at 37 °C.

The diameter of the zone around each antibiotic was measured in millimeters (mm) and calculated as the mean of three replicates. The percentage of inhibition of antibiotics was calculated using the formula described earlier by comparing it with the standard antibiotic (Penicillin) [68,69,70].

$$\mathrm{Percentage}\;\mathrm{of}\;\mathrm{Inhibition}\;(\%)=\frac{\mathrm{Zone}\;\mathrm{of}\;\mathrm{inhibition}\;\mathrm{of}\;\mathrm{test}\;\mathrm{antibiotic}}{\mathrm{Zone}\;\mathrm{of}\;\mathrm{inhibition}\;\mathrm{of}\;\mathrm{standard}}\times 100$$

### 5.5. Criteria for Bacterial Inhibition

The bacterial inhibition was carried out for *C. perfringens* isolates using the criteria mentioned (Table 7).

### 5.6. Statistical Analysis

The *C. perfringens* isolates identified from fecal samples of the control group and diseased group of animals were screened by multiplex PCR (mPCR) and the results were presented in a tabularized form. The health status-wise prevalence of *C. perfringens* types in different groups was compared by Pearson’s chi-square test (*χ*2) using SPSS software, 22.0 version (IBM, New York, NY, USA).

## Figures and Tables

**Table 1:** Carbohydrate fermentation reactions of *C. perfringens* field isolates. These isolates were placed in groups on the basis of variation in the fermentation reactions of sugars by different isolates.

Groups	Carbohydrate Fermentation															
*Clostridium perfringens*	Glucose	Mannitol	Lactose	Sucrose	Maltose	Salicin	Xylose	Arabinose	Glycerol	Cellobiose	Mannose	Melezitose	Raffinose	Sorbitol	Rhamnose	Trehalose
Group I isolates	+	−	+	+	+	−	−	−	+	−	+	−	−	−	−	+
Group II isolates	+	−	+	+	+	−	−	−	−	−	+	−	−	−	−	+
Group III isolates	+	−	+	+	+	−	−	−	−	−	+	−	+	−	−	+
Group IV isolates	+	−	+	+	+	−	−	−	+	−	+	−	+	−	−	+
Esculin positive isolates (2%)	+	−	+	+	+	−	−	−	+	−	+	−	−	−	−	+
Indole positive isolate (<1%)	+	−	+	+	+	−	−	−	+	−	+	−	−	−	−	+

+: indicates sugar fermentation and −: no sugar fermentation.

**Table 2:** Biochemical characteristics of *C. Perfringens* isolates.

Microorganism	Egg Yolk Agar		Biochemical Tests				
*Clostridium perfringens*	Lecithinase Production	Lipase Production	Gelatin Hydrolysis	Indole Production	Urease Test	Esculin Hydrolysis	Catalase Test
Group I, II, III, IV isolates	+	−	+	−	−	−	−
Esculin positive isolates (2%)	+	−	+	−	−	+	−
Indole positive isolate (<1%)	+	−	+	+	−	−	−

+: positive reaction and −: negative reaction.

**Table 3:** Multiplex PCR results for the *C. perfringens* isolates (sheep and goat).

S. No.	Toxin Gene of *C. perfringens*	*C. perfringens* Type	Positive Cases	Total Fecal Samples Collected	Animal
1.	*Alpha, epsilon, beta2*	A	65 (Diseased 42 + Healthy 23)	148	Goat
		A^β2^			
		D			
		D ^β2^			
2.	*Alpha, epsilon, beta2*	A	119 (Diseased 76 + Healthy 43)	251	Sheep
		A^β2^			
		D			
		D ^β2^			

**Table 4:** Multiplex PCR results for the *C. perfringens* isolates (healthy and diseased).

Positive Gene(s)	Isolate Population	
	Healthy (n = 68)	Diseased (n = 116)
*cpa* (Type A) *	40 (59%)	31 (27%)
*cpa*, *cpb2* (Type A, *cpb2*) *	23 (34%)	57 (49%)
*cpa*, *etx* (Type D)	3 (4%)	11 (9%)
*cpa*, *etx*, *cpb2* (Type D, *cpb2*)	2 (3%)	17 (15%)

* significantly different values in a row (*p* < 0.05).

**Table 5:** Antibiotic susceptibility testing of *Clostridium perfringens* against different antibiotics.

Antibiotics	Number of Strains of *Clostridium perfringens* with Percentage of Inhibition			
	Significant Susceptibility (>70% Inhibition)	Good Susceptibility (60–70% Inhibition)	Moderate Susceptibility (40–60% Inhibition)	Resistant (≤40% Inhibition)
Tetracycline (30 µg)	26/184(14%)	00	26/184(14%)	132/184(72%)
Teicoplanin (30 µg)	105/184(57%)	53/184(29%)	26/184(14%)	00
Enrofloxacin (5 µg)	105/184(57%)	79/184(43%)	00	00
Chloramphenicol (30 µg)	105/184(57%)	26/184(14%)	53/184(29%)	00
Rifampin (5 µg)	184/184(100%)	00	00	00
Amoxicillin (10 µg)	105/184(57%)	79/184(43%)	00	00
Penicillin G (10 µg)	184/184(100%)	00	00	00
Ceftiofur (30 µg)	184/184(100%)	00	00	00
Ciprofloxacin (5 µg)	79/184(43%)	53/184(29%)	52/184(28%)	00
Erythromycin (15 µg)	26/184(14%)	132/184(72%)	26/184(14%)	00
Norfloxacin (10 µg)	00	00	184/184(100%)	00
Linezolid (30 µg)	105/184(57%)	26/184(14%)	53/184(29%)	00
Neomycin (10 µg)	00	00	00	184/184(100%)

**Table 6:** Oligonucleotide primers for multiplex PCR detection of *C. perfringens* toxin genes.

Toxin Gene	Primers	Primer Sequence (5′–3′)	Product Size (bp)
*cpa* (α-toxin)	CPA F	GCTAATGTTACTGCCGTTGA	324 bp
	CPA R	CCTCTGATACATCGTGTAAG	
*cpb* (β-toxin)	CPB F	GCGAATATGCTGAATCATCTA	195 bp
	CPB R	GCAGGAACATTAGTATATCTTC	
*cpb2* (β_2_-toxin)	CPB2 F	AAATATGATCCTAACCAACAA	548 bp
	CPB2 R	CCAAATACTCTAATCGATGC	
*etx* (ε-toxin)	ETX F	TGGGAACTTCGATACAAGCA	376 bp
	ETX R	AACTGCACTATAATTTCCTTTTCC	
*iap* (ι-toxin)	IA F	AATGGTCCTTTAAATAATCC	272 bp
	IA R	TTAGCAAATGCACTCATATT	
*cpe* (enterotoxin)	CPE F	TTCAGTTGGATTTACTTCTG	485 bp
	CPE R	TGTCCAGTAGCTGTAATTGT	

**Table 7:** Inhibition Criteria for C. perfringens isolates.

Significant Level	Growth Inhibition
Resistant	≤40% inhibition
Moderate Sensitivity	40–60% inhibition
Good Susceptibility	60–70% inhibition
Significant Susceptibility	70% and above
